# APOE Status Modulates the Changes in Network Connectivity Induced by Brain Stimulation in Non-Demented Elders

**DOI:** 10.1371/journal.pone.0051833

**Published:** 2012-12-19

**Authors:** Cleofé Peña-Gomez, Cristina Solé-Padullés, Imma C. Clemente, Carme Junqué, Núria Bargalló, Beatriz Bosch, José Luis Molinuevo, Josep Valls-Solé, Alvaro Pascual-Leone, David Bartrés-Faz

**Affiliations:** 1 Department de Psiquiatria i Psicobiologia Clinica, Universitat de Barcelona, Barcelona, Catalonia, Spain; 2 Unitat d'Alzheimer i altres trastorns cognitius, Servei de Neurologia, Hospital Clinic de Barcelona, Barcelona, Catalonia, Spain; 3 Institut de Recerca en Cervell, Cognició i Conducta, Barcelona, Catalonia, Spain; 4 Institut d'Investigacions Biomèdiques August Pi i Sunyer, Barcelona, Catalonia, Spain; 5 Secció de Neuroradiologia, Servei de Radiologia, Centre de Diagnòstic per la Imatge, Barcelona, Catalonia, Spain; 6 Laboratori d'Exploracions Neurofuncionals, Servei de Neurologia, Hospital Clínic de Barcelona, Barcelona, Catalonia, Spain; 7 Berenson-Allen Center for Noninvasive Brain Stimulation, Beth Israel Deaconess Medical Center and Harvard Medical School, Boston, Massachusetts, United States of America; University of Montreal, Canada

## Abstract

Behavioral consequences of a brain insult represent an interaction between the injury and the capacity of the rest of the brain to adapt to it. We provide experimental support for the notion that genetic factors play a critical role in such adaptation. We induced a controlled brain disruption using repetitive transcranial magnetic stimulation (rTMS) and show that APOE status determines its impact on distributed brain networks as assessed by functional MRI (fMRI).Twenty non-demented elders exhibiting mild memory dysfunction underwent two fMRI studies during face-name encoding tasks (before and after rTMS). Baseline task performance was associated with activation of a network of brain regions in prefrontal, parietal, medial temporal and visual associative areas. APOE ε4 bearers exhibited this pattern in two separate independent components, whereas ε4-non carriers presented a single partially overlapping network. Following rTMS all subjects showed slight ameliorations in memory performance, regardless of APOE status. However, after rTMS APOE ε4-carriers showed significant changes in brain network activation, expressing strikingly similar spatial configuration as the one observed in the non-carrier group prior to stimulation. Similarly, activity in areas of the default-mode network (DMN) was found in a single component among the ε4-non bearers, whereas among carriers it appeared disaggregated in three distinct spatiotemporal components that changed to an integrated single component after rTMS.

Our findings demonstrate that genetic background play a fundamental role in the brain responses to focal insults, conditioning expression of distinct brain networks to sustain similar cognitive performance.

## Introduction

A growing body of evidence from neuropsychological, neurophysiological, and neuroimaging studies in animals and humans suggests that interactions between brain regions engaged in functional networks underlie cognitive processing and determine behavior [1], [2]. Every cognitive function and goal-directed behavior may be best identified with a certain pattern of activation of specific, spatially-distributed, but interconnected, neuronal assemblies in a particular time window and temporal order [3]. Defining network interactions is critical to understanding normal cognition and the pathophysiology of its decline [4]–[6].

Following a focal brain insult (e.g. following a stroke), or as a consequence of the alteration of function in a specific brain region (for example due to a sustained change in afferent input or efferent demand), the affected neural network adapts fluidly. This dynamic, neural plasticity can confer no perceptible change in the behavioral output of the brain, lead to changes demonstrated only under special testing conditions, or cause behavioral changes that may constitute symptoms of disease or represent paradoxical functional facilitations [7], [8].

Transcranial magnetic stimulation (TMS) provides a non-invasive technique to transiently disrupt the function of a given cortical target thus creating a temporary, “virtual brain lesion” [9], [10]. In combination with functional neuroimaging techniques, TMS provides an opportunity to study the mechanisms of dynamic network plasticity [7], [11]. TMS can be applied in trains of variable frequency and intensity to modulate the activity of a given cortical area, increase or decrease it transiently, while the subject performs a given behavior, and the brain activity associated with such behavioral activation can be measured using techniques such as functional magnetic resonance imaging (fMRI; [12]).

Genetic factors appear to critically influence network interactions and thus are likely critical contributors to the dynamic neural plasticity that allows the brain to adapt to focal disruptions [11]. While a myriad of behavioral tests may report a single, final measurement summarizing the complex interactive processes between cognition and genes, neuroimaging techniques (PET, MR, MRS, fMRI) allow us to examine more closely and immediately the biologic effects of genetic alterations [13]. Meyer-Lindenberg [14] and Rasseti & Weinberger [15] have pointed out that measures derived from brain images, in principle are closer to the underlying biology of gene action, offering an alternative target for genetic searches, by serving as intermediate phenotypes or endophenotypes. In this case, neuroimaging studies can evaluate the functional adaptation of brain activity to the controlled modulation of activity in an element of a neural network.

The apolipoprotein E (APOE) ε4 allele is the major genetic risk factor for Alzheimer's disease [16], but mounting evidence indicates that it also conditions cognitive function and brain integrity in humans across the lifespan (reviewed in [17]). Functional neuroimaging studies during cognitive tasks have revealed particular patterns of cortical activity, either in the form of decreases, increases or combination of both, among non-demented elders carrying this allele as compared with those without it [18]. These differential activation patterns have sometimes been interpreted as compensatory, particularly when similar cognitive performances can be observed among genetic subgroups, though sometimes they appear related to suboptimal cognitive function in ε4 carriers [19]–[24]. Importantly, APOE status has recently been shown to modulate large-scale networks as identified by the analysis of low-frequency fMRI-BOLD fluctuations. Particularly, the activity of the so called *default mode network* (DMN), often affected in clinical and preclinical AD [25], [26], appears already compromised in non-demented elders carrying this allele during memory encoding tasks by virtue of failures to deactivate posteromedial regions [27]–[29].

The aim of the present study was to investigate whether the genetic background for APOE status differentially modulates the brain response to the induced effects of rTMS on functional large-scale networks, and to determine the relation to behavioral changes and underlying gray matter integrity. To our knowledge this is the first study combining rTMS and fMRI to demonstrate that individual differences in a relevant human genetic variant among the elder determine brain response to focal brain disruption.

## Methods

### Subjects

Twenty subjects older than 50 years (mean age: 66.95, SD: 9.42; female: 15: see below table 1) were recruited from a primary health center (CAP Castellar del Vallès) and the Alzheimer's Disease and Related Disorders Unit at the *Hospital Clinic de Barcelona*. Despite the fact that all subjects complained of memory problems, they all were free of dementia and depression according to clinical and neuropsychological assessments. Dementia was ruled out using a clinical and neuropsychological examination including measures of global cognitive function (MMSE ≥24), language (Token test), praxis (imitation and performance to command), gnosis (Poppelreuter's embedded figures and Luria's watches) and abstract reasoning (WAIS III Similarities subtest). Depression was ruled out through a Hamilton Depression Scale cut-off score of 15. Included cases were restricted to those exhibiting impairments in memory domain since all of them scored −1 SD below standardized age-matched norms in at least one of the following memory tests: Rey Auditory Verbal Learning Test (RAVLT) and Visual Reproduction of the Wechsler Memory Scale Revised (WMS-R). The Ethics Comitee of the Hospital Clinic de Barcelona, Spain, approved this study and all subjects gave their written informed consent to participate.

**Table 1:** Demographic and global cognitive characteristics of the participants.

	ε4 carriers	ε4 noncarriers	Statistical values	P values
**Age**	66.77 (9.67)	67.09 (9.68)	t = 0.07	0.93
**Years of formal education**	6.11 (3.48)	7.45 (2.80)	U = 38.5	0.41
**MMSE**	26.33 (2.34)	26.63 (1.91)	t = 0.31	0.75
**Gender (M/W)**	(6/3)	(9/2)	*χ* ^2^ = 0.61	0.15
**Inferred IQ (Vocabulary WAIS-III)**	50.89 (8.95)	51 (10.89)	t = 0.02	0.98
**Verbal Memory (RALVT)**	7.73 (2.28)	6.25 (3.89)	t = 1.04	0.31
**Visual Memory (VR-WMS-R)**	10.88 (5.51)	10.33 (7.23)	t = 0.16	0.88

Note: Values are given in mean (SD). MMSE^a^, mini-mental state examination. (M/W) M, male; W, women. WAIS: Wechsler Adult Intelligence Scale. RAVLT: Rey-Auditory Verbal Learning test (delayed recall). VR-WMS-R: Visual Reproduction, Wechsler Memory Scale Revised (delayed recall). t = Student's test. U = U-Mann Whitney test.

### Apolipoprotein E

Genomic DNA was isolated from peripheral blood leukocytes. At the APOE locus, the polymorphism of the three common genetic variants, ε2, ε3 and ε4, due to Cys-Arg substitutions at amino acid positions 112 and 158 was analyzed. The polymerase chain reaction was used to amplify the alleles of the APOE gene as described elsewhere [30]. In our twenty subjects, 9 were carriers of the ε4 variant (6 ε3/ε4, 2 ε2/ε4 and 1 ε4/ε4) and 11 were noncarriers (10 ε3/ε3 and 1 ε2/ε3).

### MRI acquisition, baseline fMRI session and memory encoding assessment

All scans were obtained on a GE Signa 1.5T (General Electric, Milwaukee, WI). High-resolution T1-weighted images were acquired for anatomical identification with a FSPGR three-dimensional sequence (DICOM format, TR/TE = 12/5.2; TI 300 1 nex; FOV = 24×24 cm; 256×256 matrix). Whole-brain volumes were acquired in an axial plane yielding contiguous slices with slice thickness of 1 mm. Functional images were acquired using a T2* weighted gradient-echo planar imaging (TR = 2000 ms; TE = 40 ms; FOV = 24×24 cm; flip angle of 90°). Twenty axial slices were obtained for each brain volume with a slice thickness of 5 mm and a gap of 1.5 mm.

For fMRI, we used a block design with alternating rest and experimental conditions (five blocks each). The task required encoding and learning of visually-presented face-name pairs. Before the fMRI session, subjects learned 2 face-name pairs, which were used later as control stimuli (control condition). During the experimental condition subjects were presented 10 new face-name pairs to be learnt during the scanning. The duration of each stimulus (face-name pair) was 2 s and the inter-stimuli period was 1 s. The whole experiment lasted approximately 300 s (30 s per block, 150 s for each condition). Following the fMRI session participants were assessed for recognition memory of the 10 face-name pairs learnt. For this purpose, individuals were shown 10 printed photographs as well as 10 written names and were instructed to pair each name with the corresponding face as they remembered from the fMRI session. Only the stimuli used in the experimental blocks were presented during the associative memory task, and thus, only correct/incorrect face–name matches were recorded as responses. The maximum score for this task was 10 (all names correctly matched with the corresponding face).

### rTMS

rTMS was applied between a first and second fMRI examination. A MAGSTIM SUPER® stimulator and a double-cone coil were used. The intensity of TMS pulses was set at 80% of the individual's motor threshold with the intersection of the double-cone coil positioned over the left primary motor cortex. For prefrontal cortex stimulation, the TMS coil was moved anteriorly approximately 5 cm and centered in the vertex (interhemispheric fissure) to affect prefrontal cortex bilaterally. Ten rTMS trains lasting 10 s each were delivered during a 5-minute period using a frequency of 5 Hz (total number of pulses 5000). Specifically, every 30 s, subjects were given 10 s of rTMS followed by a 20 s rest period. Previously we showed that in this form, active rTMS exerts significant effects both at the cognitive and brain activity levels, whereas sham rTMS does not result in any observable effects [31]. Therefore, in the present study all 20 individuals received active rTMS and group comparisons were based on the analysis of brain networks expression on the basis of the APOE status.

### Second fMRI session and memory assessment

Immediately after the rTMS, subjects underwent a second fMRI examination. The average time elapsed between the end of the first and the beginning of the second fMRI was 9.99 min±1.99 (SD). We used an equivalent 10 face-name pairs learning task that was counter-balanced with the one administered during the first fMRI session. Once the scanning procedure was finished, participants were tested for memory recognition as in the baseline fMRI session.

### Statistical analyses of non-imaging data

Demographic and cognitive data analysis was implemented in SPSS v.14. Chi-squared test was used to compare gender distribution between groups (APOE ε4 carriers versus noncarriers). For continuous variables, assumptions for normality and homocedasticity were tested for all cognitive and demographic variables. Considering these observations, all comparisons were performed using parametric tests (Student's t test and ANOVA) except those concerning the years of education variable for comparing both genetic subgroups that were achieved using the U Mann-Whitney test. All tests were two-tailed and statistical significance was set at p<0.05. A two-way ANOVA was undertaken to examine whether rTMS exerted a distinct effect between genetic subgroups across the two memory examinations using genetic subgroup as the between-subject factor and moment of the memory evaluation (first or second fMRI) as within-subject factor.

### fMRI-ICA analysis-strength of activity and network correlation analyses

In order to investigate the differences within and between groups in the strength of activation corresponding to the encoding phase in the fMRI-task we performed a group tensor-ICA (T-ICA) merging all the sessions (pre and after rTMS) and groups (ε4-carriers and noncarriers). The group T-ICA allows the determination of the brain areas (networks) common to all subjects for later group comparison. Image preprocessing and T-ICA analyses were performed using MELODIC [32] version 3.10, implemented in FSL software (http://www.fmrib.ox.ac.uk/fsl/). The preprocessing steps included motion correction, skull stripping, spatial normalization to the Montreal Neurological Institute (MNI) template (resampling voxel size = 3 mm×3 mm×3 mm), spatial smoothing (FWHM = 6 mm), and grand-mean scaling. Functional data was temporally band-pass filtered using a high pass filter of 60 s to reduce the possible physiologic noise. Moreover, the possible nuisance on the data induced by movements inside the scanner was regressed out. Finally, a tensor-ICA decomposition of the preprocessed images was performed.

The brain maps (networks) corresponding to the activation-encoding period and deactivation-rest period were used for network correlation analysis and cortical thickness analysis (explained in detail below). Concerning the network correlation analyses, the mean BOLD-time series corresponding with the brain areas activated during the encoding phase as well as those activated during the rest phase were extracted and correlated for each subject. This procedure resulted in r-Pearson values between the time course of activation and deactivation periods examined across sessions (pre/post rTMS) and groups (APOE ε allele carriers/noncarriers). The Pearson correlation values were transformed to z-scores (using Fisher z-transform) and then averaged over all subjects for later statistical group comparison. Concretely, a paired t test was performed for each group to test the possible correlation changes occurred after administering rTMS.

### fMRI-ICA analysis-Spatial pattern correlation analyses

We investigated quantitatively and qualitatively the changes in the spatial patterns of the networks after stimulation. Regarding the quantitative analyses the comparisons amongst these networks were done using ICA and the spatial pattern correlations implemented both in the GIFT software ([33]; http://icatb.sourceforge.net/). After image preprocessing steps were completed, we performed the group spatial ICA.

First, we performed one group spatial ICA for each fMRI session (pre/post TMS) and group (ε4 carrriers/noncarriers), resulting in 4 independent group spatial ICA comparisons. Specifically, after subject-wise data concatenations, ICA was performed in three stages: i) using a principal component analysis (PCA) we performed a data reduction (2 steps) of each subject's fMRI data to the number of components previously estimated by the minimum description length (MDL) criterion [34]; ii) application of ICA algorithm (infomax); and iii) back reconstruction for each individual subject's data generating time courses and spatial maps [33]. The degree of correlation of a given voxel's fMRI signal with the time course of the component was represented by the Z score within the spatial map [35].

Second, we performed a temporal correlation analysis (TCA) between those networks that had a significant correlation with the encoding and rest conditions, respectively. Moreover, in the former condition we only considered those networks that exhibited a significant positive correlation between the performance and the beta weights of each subject extracted from the networks synchronized with encoding phase (given by TCA). Beta weight accounts for the particular contribution of each subject (weight) in the group correlation value obtained by TCA. This procedure was done in order to assure that the networks taken into consideration were informative and significant for the encoding phase as well as for performance. Finally, we were able to perform spatial pattern correlations amongst the selected networks.

### MRI analysis of cortical thickness and volumetric segmentation procedures

To investigate whether putative distinct functional brain responses to rTMS observed between APOE groups were related to underlying gray mater atrophy we used the IC (thresholded at z = 2.3) corresponding to active areas when subjects were encoding new faces to create a binary mask for subsequent cortical thickness analysis. This binary mask was mapped for each of the subjects in order to extract the cortical thickness and grey matter volume of each hemisphere. With this procedure we assured that the areas of comparison related with the activity were the same for all the subjects. These values were used for further comparison between groups. Additionally, we also performed a whole brain analysis of the entire cortical mantle, in order to evaluate possible effects of cortical atrophy on cortical networks.

To carry out cortical thickness analysis we used Freesurfer version 5.0 software (http://surfer.nmr.mgh.harvard.edu/). Technical details of the procedures to obtain the CTh maps for posterior analysis have been previously and fully described elsewhere [36]. The procedures for the measurement of CTh have been validated against histological analyses [37] and manual measurements [38]. Freesurfer morphometric procedures show good test-retest reliability across scanner manufacturers and field strengths [39].

We smoothed the reconstructed and registered individual CTh maps of complete cortical models with a surface-based Gaussian kernel of 10 mm full width at half maximum (FWHM). Comparison analyses between ε4 carriers and noncarrier groups were based on vertex-by-vertex general linear modeling (GLM) of the CTh, with gender and age introduced as nuisance factors. We used Monte Carlo simulation (10.000 repetitions) to calculate cluster-wise statistical significances corrected for multiple comparisons.

Additionally, we performed ANCOVA on hippocampus volume measurements extracted from Freesurfer comparing ε4 carriers and noncarriers, with covariates of gender and age as possible confounding factors. We corrected the hippocampus volume using a previously validated estimate of the total intracranial volume (eTIV) [40].

## Results

### Group comparisons and behavioral effects of rTMS

APOE ε4-carriers and noncarrier groups were comparable in terms of age, gender distribution, years of formal education and cognitive status, including verbal and visual memory assessments (see table 1). Consistent with our previous report [31], we observed an improvement of recognition memory scores during the second fMRI, following rTMS for the whole sample. Specifically, when we tested for the interaction of group (APOE status) x time of evaluation of memory performance (pre or after rTMS), it was not significant, suggesting similar memory changes across genetic subgroups (table 2).

**Table 2:** Measures of recognition memory before (pre) and after (post) rTMS.

	PRE	POST	Statistic	P values
**Whole sample**	4.15 (2.94)	5.75 (2.99)	t = 2.32	0.03
**ε4 carriers ε4 noncarriers**	TMS_pre_ = 4,22 (3,11) TMS_pre_ = 4,09 (2,94)	TMS_post_ = 5,67 (3,35) TMS_post_ = 5,82 (2,82)	F = 0.76	0.39

Values are given in mean (ranges). t = repeated measures t test comparing pre vs post rTMS in the whole sample. F = results of the interaction value of the two-factor ANOVA using genetic subgroup as the between subject factor and moment of the evaluation (PRE vs POST) as within subject factor.

### Common fMRI networks across sessions and groups

**Figure 1:**
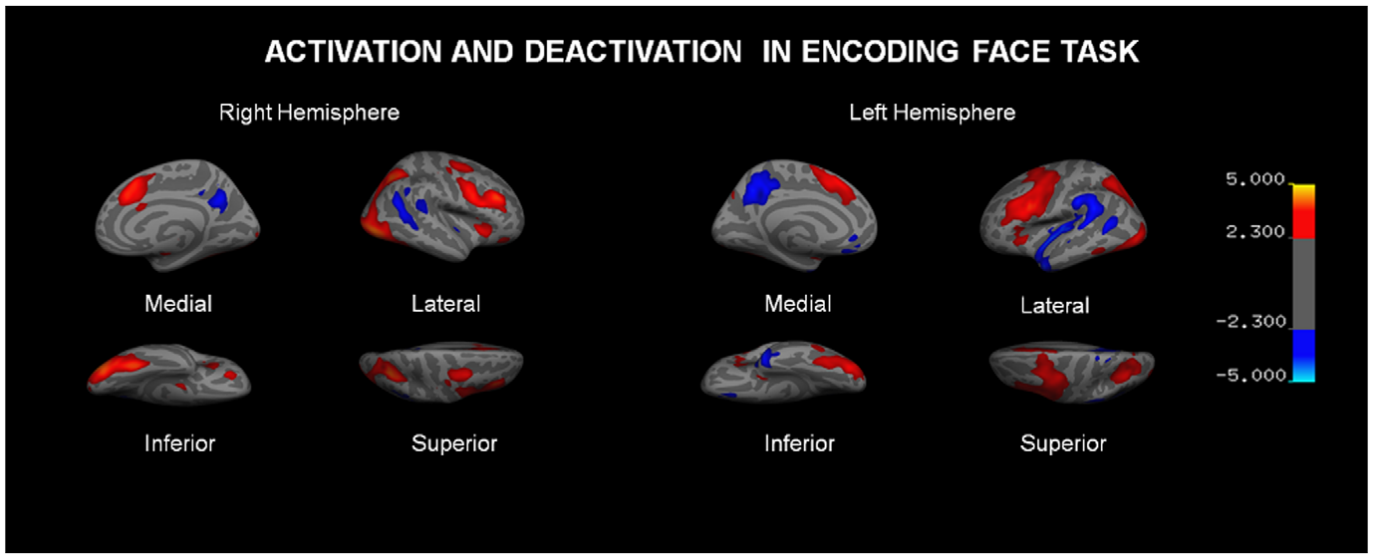
Independent components (IC, networks) that were highly correlated with the encoding period and performance (ATBRP = activation-task brain related pattern). Networks are separated for each condition (pre-post TMS) and group (ε4 carriers-noncarriers). Coordinates are given in MNI space. Intensity values are thresholded at z = 2.3. Brain areas of each network are fully described in table 3.

The T-ICA analysis identified a network that was significantly (p<0.01) related to the encoding of new faces across sessions (pre/post rTMS) and groups (ε4 carriers/non-carriers). In figure 1, the areas belonging to this network, and active during the encoding period, are depicted in red, while the areas active in the repeated faces blocks (control condition) are shown in blue. The main regions active during encoding in both groups comprise primary visual and associative regions, temporoparietal areas including the fusiform gyrus, the parahippocampus and the hippocampus, as well as motor, premotor and prefrontal areas. On the other hand the areas showing greater activity during repeated stimuli (i.e. or deactivated during encoding of novel information) align with the anatomical descriptions of the DMN, mainly including anterior and posterior midline structures as well as the inferior parietal cortex.

APOE had a small effect on overall activity level. The analysis revealed only a significant differential activity (p<0.05) between sessions (pre-post) in the ε4 noncarriers group, where higher activity was observed in the second fMRI examination, after TMS administration.

The ε4-noncarrier group exhibited a more pronounced pattern of anticorrelation between the time series of activation and deactivation areas at baseline (z_PRE-RTMS_ = 0.09 vs z_PRE-RTMS_ = −0.05, for ε4 carriers and noncarriers, respectively). However, this difference did not reach statistical significance (p = 0.57). Similarly, paired t-test analysis for each group did not revealed any significant change, though both groups showed increased anticorrelation after rTMS (z_POST-RTMS_ = −0.02, for ε4-carriers; z_POST-RTMS_ = −0.11, for noncarriers).

### Group x session activation of performance-related patterns differences

**Figure 2:**
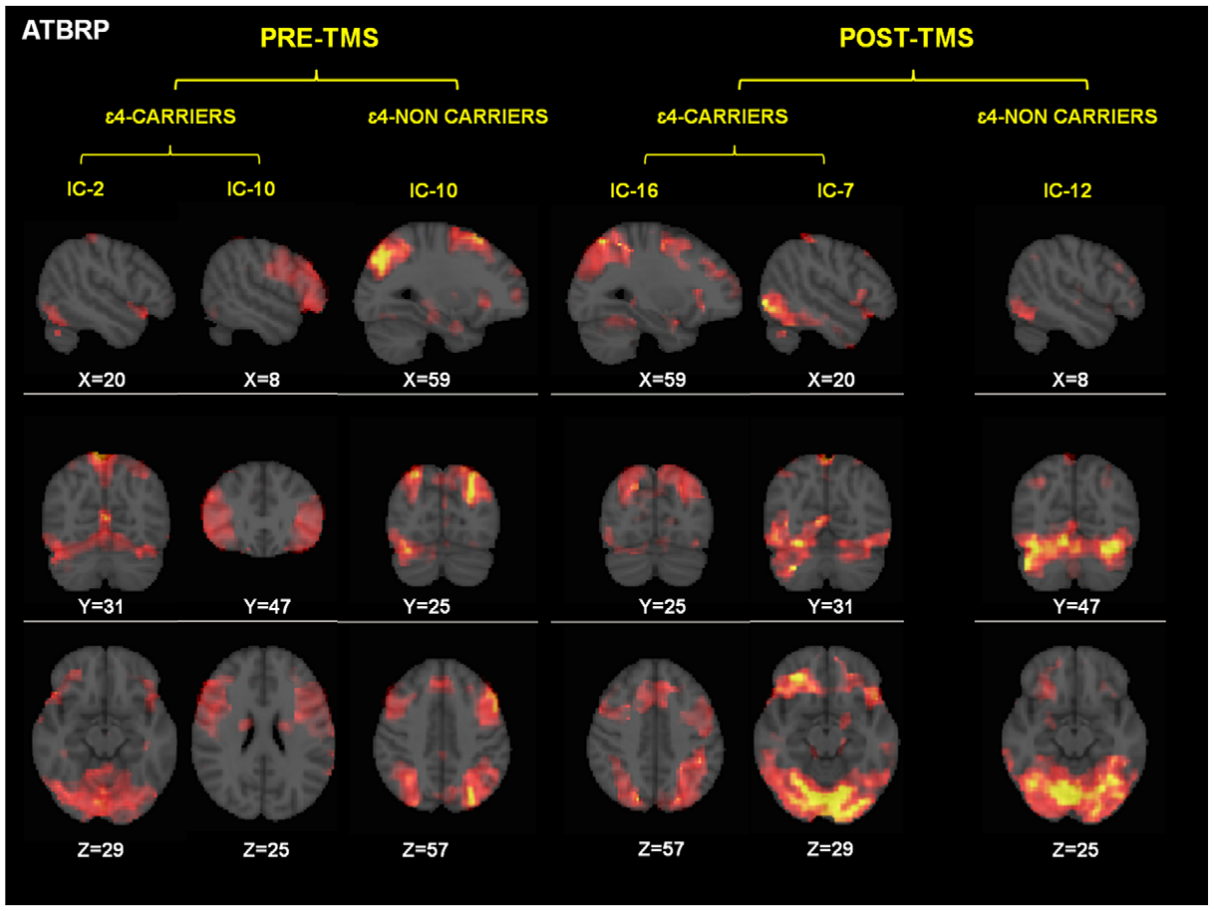
Independent components (networks) that were significantly correlated with the deactivation period (DTBRP = deactivation-task brain related pattern). Networks are separated for each condition (pre-post TMS) and group (ε4 carriers-noncarriers). DTRP = Dectivation Task Related Patterns. Coordinates are given in MNI space. Intensity values are thresholded at z = 2.3. Brain areas of each network are fully described in table 4.

Figure 2 and table 3 depict different sets of independent components that were significantly correlated with the blocks of encoding of new face-name pairs, as well as with subsequent memory performance in each genetic subgroup.

**Table 3:** Brain Networks related with encoding and subsequent memory performance.

APOE-ε4	rTMS	IC	Brain Areas
**Carriers**	Pre	2	Primary and associative visual cortex (BA 17, 18, 19), Posterior Cingulate (BA 30), Fusiform gyrus (BA 37), Orbitofrontal area (BA 11), Superior Parietal (BA 7), Temporopolar area (BA 38)
	Pre	10	Middle frontal gyrus (BA 9, 46), Inferior prefrontal gyrus (BA 44, 45, 47), Premotor and Motor Cortex (BA 4,6), Left Parahipocampal formation (BA 30L)
	Post	16	Associative visual cortex (19), Inferior and superior parietal cortex (7,40), Anterior cingulate cortex (32,33), Premotor cortex (BA 6), Middle frontal gyrus (BA 46, 47), Hippocampus, Fusiform gyrus (BA 37)
	Post	7	Primary and associative visual cortex (BA 17 18, 19), Fusiform gyrus (BA 37), Superior parietal cortex (BA 7), Temporopolar area (BA 38), Inferior prefrontal gyrus (BA 47), Orbitofrontal area (BA 11), Right Hippocampus,
**Noncarriers**	Pre	10	Superior parietal cortex (BA 7), Left primary and associative visual cortex (BA 18, 19), Inferior prefrontal gyrus (BA 44), Middle frontal gyrus (46, 8), Premotor cortex (BA 6), Fusiform gyrus (BA 37), Hippocampus and Parahipocampal formation
	Post	12	Primary and associative visual cortex (BA 17,18,19), Fusiform gyrus (BA 37), Superior parietal cortex (BA 7, 5), Left Orbitofrontal area (BA 11L), Hippocampus

Regarding the pre-TMS condition, ε4 bearers exhibited two components, which encompassed mainly the fusiform gyrus and visual areas (IC-2 in table 3) as well as bilateral frontal areas (IC-10). In contrast, ε4-non carriers only presented one single component (IC-10) which mainly included frontoparietal and fusiform areas.

Following rTMS among ε4-carriers a new frontoparietal component emerged (IC-16), whereas the fusiform gyrus component (IC-7) was still observed after stimulation, albeit more active than at baseline (IC-2). Therefore, among ε4 carriers the brain network IC-16 exhibited a significant correlation with task performance after brain stimulation showing strikingly similar spatial configuration as the one observed in the non-carrier group prior to stimulation (see figure 2 and table 3).

On the other hand, in the ε4-non carrier group, after TMS a similar topographical and unique component as before stimulation could be identified (IC-12) albeit in this case it showed a decrease of brain activity in the DLPFC and an increase in visual associative regions such as the fusiform gyrus, lingual gyrus, and visual areas was significantly associated with task time course and performance (see figure 2 and table 3).

### Group x session differences in deactivation task-related patterns

**Figure 3:**
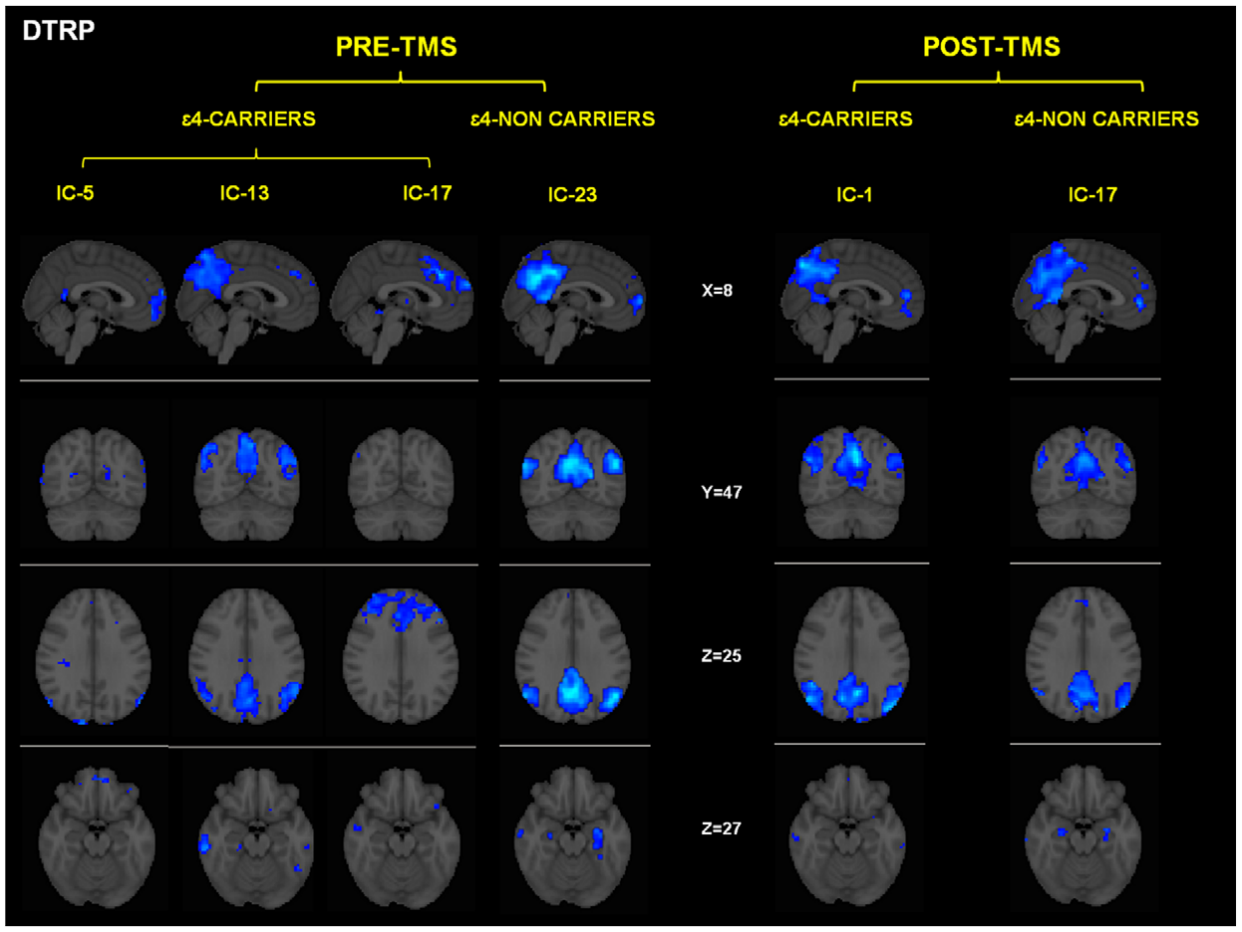
Effects of rTMS in the DMN present in deactivation task-related networks. While both groups exhibited increased temporal correlations between the timecourse of this network and rest condition after rTMS, its activity (intensity of the expression) clearly diverged. In the bar graphs it is shown that DMN activity decreased for ε4 non-carriers whereas increased for the ε4-carriers. Corr: Correlation values (r-Pearson) between the timecourse of each network and the ‘resting condition’. Intensity values are thresholded at z = 2.3. Coordinates are given in MNI (x = −4, z = 26). A.U = arbitrary units.

The functional networks that showed a significant correlation between their temporal fluctuation and the condition where no learning occurred (i.e. ‘control condition’) are depicted in figure 3. It is important to note here, that all these areas showed decreased activity during the encoding phase and consequently might be interpreted as patterns of deactivation. In both genetic groups the common spatial pattern is similar, encompassing midline frontal, precuneus and lateral inferior parietal (see table 4). These brain regions have close anatomical correspondence with the well-characterized default-mode network (DMN) [41], [42].

**Table 4:** Brain Networks related with deactivation.

APO-ε4	rTMS	IC	Brain Areas
**Carriers**	Pre	5	Anterior prefrontal cortex (BA 10), Orbitofrontal cortex (BA 11), Inferior prefrontal gyrus (BA 47), Retrosplenial cingulate cortex (BA 26, 29), dorsal anterior cingulated cortex (BA 32), Primary and secondary visual cortex (BA 17 18), Angular gyrus (BA 39).
	Pre	13	Inferior and superior parietal cortex (BA 39, 40, 5, 7), Secondary visual areas (BA 18), Posterior and anterior cingulate cortex (BA 30, 23, 32), Middle temporal gyrus (BA 21), Anterior prefrontal cortex (BA 10), Precuneus, Left Parahippocampal
	Pre	17	Anterior prefrontal cortex (BA 10), Dorsal anterior cingulate (BA 32), Middle prefrontal cortex (BA 9, 46), Angular gyrus (BA 39)
	Post	1	Inferior and superior parietal cortex (BA 39,40,5,7), Primary and secondary visual cortex (BA 17,18), Posterior and anterior cingulate cortex (BA 23, 30, 32), Retroesplenial cortex (BA 29), Fusiform gyrus (BA 37), Middle and inferior temporal gyrus (BA 20, 21), Anterior prefrontal cortex (BA 10), Parahippocampal Formation, Precuneus,
**Noncarriers**	Pre	23	Inferior and superior parietal cortex (BA 39,40,7,5), Primary and secondary visual cortex (BA 17,18), Posterior and anterior cingulate cortex (BA 23,30,32), Retroesplenial cortex (BA 29), Precuneus, Fusiform gyrus (BA 37), Middle temporal gyrus (BA 21), Anterior prefrontal cortex (BA 10), Hippocampus, Parahippocampal formation,
	Post	17	Inferior and superior parietal cortex (BA 39,40,7,5), Primary and secondary visual cortex (BA 18), Posterior and anterior cingulate cortex (23,30,32), Retroesplenial cortex (BA 29), Precuneus, Fusiform gyrus (BA 37), Middle temporal gyrus (BA 21), Anterior prefrontal cortex (BA 10), Hippocampus, Parahipocampal formation,

Despite similar topographic patterns, there were however relevant differences between the genetic groups in the deactivation patterns, as APOE ε4-carriers exhibited three separate independent components (IC-5, IC-13, IC-17 in table 4), while among noncarriers a single component was present (IC-23, see figure 3 and table 4).

The administration of rTMS had no observable effect on the number and general characteristics of the deactivation components among the ε4-noncarrier group, whereas in the ε4-carriers the numbers of components were reduced and integrated into a single component (IC-17) after stimulation. This component (IC-17) was highly similar (spatial correlation = 0.5) to the component observed before and after rTMS among ε4-noncarriers. Despite both groups showed deactivations within areas of the parahippocampal formation, deactivation of the proper hippocampus within the ε4-noncarrier group was observed both pre and post rTMS, whereas it was not present among the ε4-carriers at any of the timepoints.

**Figure 4:**
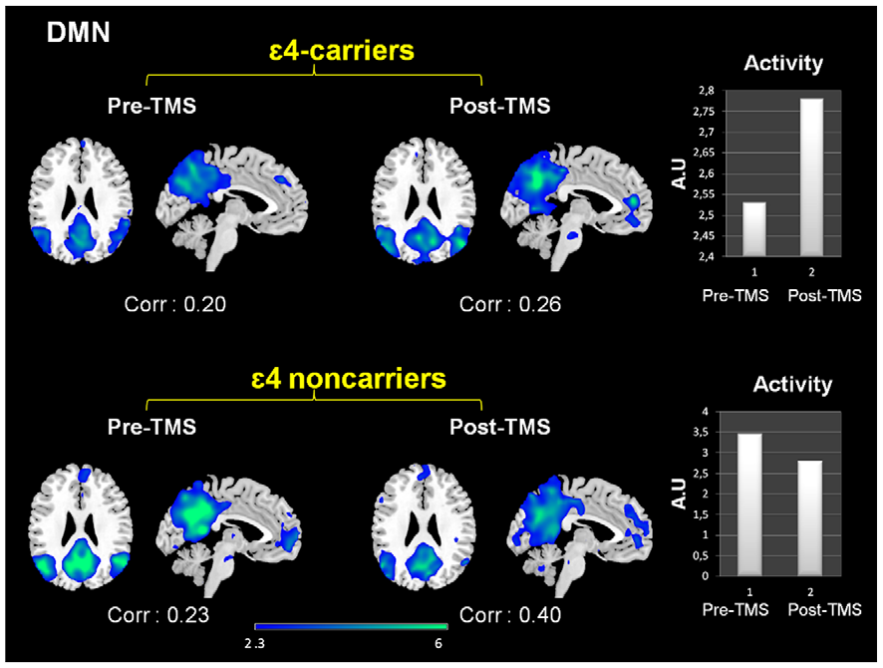
Effects of rTMS in the DMN present in deactivation task-related networks. While both groups exhibited increased temporal correlations between the timecourse of this network and rest condition after rTMS, its activity (intensity of the expression) clearly diverged. In the bar graphs it is shown that DMN activity decreased for ε4 non-carriers whereas increased for the ε4-carriers. Corr: Correlation values (r-Pearson) between the timecourse of each network and the ‘resting condition’. Intensity values are thresholded at z = 2.3. Coordinates are given in MNI (x = −4, z = 26). A.U = arbitrary units.

When looking at the intensity of deactivation by performing cross correlations between each network and a template of DMN (provided within the GIFT software), it was evident that among ε4-noncarriers the intensity of expression of the DMN was decreased subsequently to brain stimulation, whereas it was increased for APOE ε4 carriers. These results reflect enhanced connectivity between the components of the DMN areas after TMS in this group (see figure 4).

### Cortical thickness and morphology analysis

APOE ε4-carriers exhibited reduced CTh in different areas of the right hemisphere (see table S1). However, the cortical thickness comparison of the ROIs corresponding to the functional activation of left and right hemispheres (figure S1) revealed no differences between genetic groups. When comparing differences in hemispheric gray and white matter volumes as well as the hippocampus, ε4-carriers showed reduced volumes and increased CSF spaces, but differences did not reach statistical significance (tables S2 and S3). Altogether these observations suggest that differences in cortical or subcortical anatomy cannot account for the particular functional activity and connectivity differences observed in the present study between genetic subgroups.

## Discussion

The main finding of the present study is that APOE status, the most relevant genetic factor associated with Alzheimer's disease and age-related cognitive decline, differentially modulates the cerebral responses to brain stimulation during a memory encoding task, even if no differences could be observed at a cognitive level at any stage between genetic subgroups. To our knowledge present results offer first evidence demonstrating the influence of genetic factors onto the brain adaptive mechanisms to focal brain disruption as measured by functional neuroimaging.

Previous reports have been published demonstrating that the effects of rTMS can be affected by phenotypic differences associated with genetic variations causative of several neurological conditions such as cerebellar ataxia-I (spinocerebellar ataxia) [43], [44] or hereditary spastic paraplegia (HSP) [45]. Greater improvements following rTMS administration in depressive patients [46] have been reported depending on the genetic background of the serotonin transporter (5-HTTLPR) and receptor (5-HT1A) genes. Also, homozygous subjects for the Val allele (but not *Val/Met* and *Met/Met* genotypes) of the val66met brain-derived neurotrophic factor gene (BDNF) showed a significant increase in mean first dorsal interosseous map area, MEP amplitude and map volume after the performance of 30 min fine-motor exercises [47]. Similarly, *Met* allele carriers of the BNDF have been more recently shown to exhibit reduced response to theta-burst stimulation (TBS) by TMS [48], therefore revealing the effect of genetic variations associated with modification of use-dependent plasticity in the motor cortex.

In the present study we combined rTMS with behavioral, genetic and functional imaging data. The most striking result is that APOE ε4 carriers showed a different pattern of brain activation and deactivation during the encoding task than noncarriers. However, after rTMS these patterns became much more similar, due to a preferential impact of rTMS on patterns of brain activity in APOE ε4 carriers, since brain activity in noncarriers was little modified.

At baseline, before rTMS, both groups show enhanced activity in brain networks involving frontal, associative visual (i.e. fusiform) MTL regions (hippocampus, parahippocampus) as well as the cerebellum. All these regions have been previously related to visual memory encoding in seminal studies employing face-name associative memory tasks [49], [50]. Despite there were no significant differences in this global component (all subjects all sessions) across groups, in the ε4-noncarrier group the frontal component involved also the superior parietal lobe in a patterns that topographically resembled the ‘frontoparietal’ network generally related to attentional and working memory processes [35], whereas individuals bearing the ε4 allele recruited more inferior frontal lobe areas, the orbitofrontal cortex and the anterior cingulate region.

We then analyzed the pattern of brain activity separately for each genetic group and restricted to the areas that covaried with performance. Here, previous to the administration of rTMS, the ε4-carriers group exhibited two networks, which were present in the encoding, whereas the ε4-noncarriers exhibited activity integrated into one single network. Following rTMS, in the ε4-noncarrier group a pattern of activity restricted to the fusiform gyri and visual areas was evident, gathered into one single network. On the other hand, after rTMS, two networks increased its expression in the ε4-carrier group: The first one mainly included the fusiform gyri and the second a component that was not observable in the pre-rTMS condition within this group but which had a higher resemblance with the network present before rTMS in the ε4-noncarriers (the fronto-parietal network).

Altogether, present findings show that rTMS may have caused a redistribution of neural network dynamics, enhancing activity in areas that are commonly related with the encoding task but also favoring cortical activation in regions prone to modulate their activity during memory encoding depending on the individual genetic characteristics of subjects. Increased activity in cortical frontal and posterior regions has been repeatedly reported in the literature during distinct types of memory tasks among APOE-ε4 carriers and related to executive-mediated compensatory brain mechanisms in the face of cognitive challenges [19], [20], [22], [51]–[53]. Increased pattern of brain activity within the fusiform has also been observed in young samples during memory encoding demands [54]. Therefore, it appears that rTMS brought out patterns of activity previously reported, possible revealing established compensatory brain activity strategies.

Main effects were expected in the prefrontal region (since it was the site of stimulation), but we had no specific anatomical predictions for other areas, given that APOE influence on associative areas of *all* cerebral lobes has been reported (e.g. [19], [20], [22], [55]. Since the effects of rTMS captured by fMRI are not restricted to the local area where it is applied [11], [12] we also investigated the possible rTMS effects on network dynamics when subjects viewed passively and repeatedly an already known pair face-name (i.e. the ‘deactivation network’). Shulman et al. [41] showed that in these conditions, rest phases of an ongoing task, a set of areas are typically active regardless of the nature of the task, the so-called DMN. Interestingly, Alzheimer disease patients, but also pre-symptomatic individuals, show less robust deactivation of core areas of the DMN during memory encoding tasks [27], [29], [56]. Besides the fact that metabolic dysfunctions, amyloid deposition or atrophy [8] may directly impact the posterior DMN, the alteration of this network may be accounted for by disruptions in the functional connectivity between anterior and posterior core regions [57].

Previous studies in elders, have demonstrated that during cognitive processing lesser deactivations are found with decreasing task demands particularly in the posterior node of the DMN, and that these functional changes correlate with task performance [58]–[60]. These findings therefore suggest that for a given level of task difficulty individuals who are cognitively more efficient will require reduced posteromedial deactivations. In our study, among the non-carrier group and following rTMS there was little change in terms of the topography of the deactivation pattern, but there was a relative reduction of the connectivity of these posteromedial regions after stimulation. As these changes emerged within the context of a cognitive amelioration, present observations reinforce the idea that these subjects when faced the second time with an equivalent task could employ more efficient brain resources.

In contrast to non-carriers, the pattern of connectivity of the DMN changed dramatically in ε4-bearers after TMS. DMN alterations among APOE ε4-bearers have been reported previously, both in resting-state and as attenuated deactivation areas [18], [28], [54], [58], [61], [62]. After rTMS DMN activity changed from three separate networks to a more robustly connected single one, resembling the one observed among non-carriers. Still, a lack of connectivity between the DMN and the hippocampus among ε4-bears was observed both before and after rTMS. Previous reports are consistent in observing abnormal activity of this region among ε4-bears during visual encoding tasks [52], [62] including deviant patterns of connectivity [18], [23]. Hence it is conceivably that the activity of this region was relatively permeable to rTMS effects in these individuals.

The functional changes observed in ε4-carriers after rTMS may be related to a genetic modulation of APOE on brain plasticity mechanisms. The effects of non-invasive brain stimulation techniques, including rTMS and transcranial direct current stimulation have been reported to modulate synaptic and plastic brain mechanisms that can be captured by imaging techniques (i.e. [63], [64]). Plasticity is an intrinsic capacity of the human brain [7], which changes across lifespan depending on individual characteristics such as lifetime exposure to particular environments and the genetic background [11]. APOE is involved in neural development and repair functions [65], [66] and APOE-ε4 selectively impairs synaptic plasticity and NMDA receptor phosphorylation by Reelin, a regulator of brain development and modulator of synaptic strength [67]. Furthermore, APOE ε4 is related to higher fibrillar β-amyloid deposition according to PiB findings [68] as well as to lower (i.e. more pathological) Aβ1-42 CSF concentrations [69] in non-demented individuals. In turn, distinct Aβ species, particularly soluble oligomers, are known to compromise synaptic plasticity, inducing long term potentiation (LTP) deficits and compromising cognitive functions (reviewed in [70]). Finally, increased Aβ deposition both in soluble and fibrillar forms can be observed in response to increasing neural activity, even in young animals [71]. An increase in aerobic glycolysis related to elevated synaptic activity occurs in DMN areas [72] and high-sustained neural activity in this network may be a characteristic of APOE ε4-carrier subjects from their young adulthood [54]. Therefore, it is possible that a more compromised fundamental plastic brain mechanisms among APOE ε4-carriers may represent an underlying mechanism leading to a distinct expression of brain networks as well as to a differential response of these to brain stimulation.

Complimentarily, the differential patterns of activation observed among our APOE ε4-carriers may reflect a non-optimal use of cortical networks compatible with the concept of dedifferentiation (i.e. loss of highly specialized neural systems during specific tasks; [18], [73]). Dedifferentiation occurs with advancing age, and age is a relevant modulating factor for functional brain responses in regions such as the hippocampus [54], [61], [74] among ε4-carriers. Dedifferentiation has been reported to occur in the fusiform area during face perception [75], a critical region activated in our face-name task with different patterns in response to rTMS in both groups).

An obvious limitation of the present study is that we did not include equivalent genetic subgroups receiving sham stimulation. Despite further studies with larger samples may be need to address a possible differential genetic response to placebo stimulation, it seems unlikely that sham rTMS would exert differential effects in subjects depending on the APOE status or that practice effects on memory encoding rather than as a consequence of rTMS explain our findings. This is supported by an exploratory analysis performed in a group of 4 ApoE ε4 carriers and 15 non-carriers receiving sham stimulation reflecting no changes both at the behavioral level or in the expression of networks when comparing the data before and after placebo stimulation (data not shown). The fact that we did not use a neuronavigation system to deliver rTMS prevented us from establishing more concrete anatomical hypotheses. Further studies with larger samples and more refined MRI-based TMS coil positioning procedures should overcome these limitations.

In summary, we demonstrate that genetic factors determine the pattern of compensatory plastic changes following a focal brain disruption (in this case induced by rTMS) and thus ultimately influence its behavioral consequences. The present findings may have profound implications for clinical neurology as they suggest that genetic factors play a crucial role in the inter-individual differences in recovery and the risk of development of disability following a focal brain insult. Although the exact neurophysiologic mechanisms that regulate the connectivity amongst the functional networks and particularly the DMN are very difficult to disentangle, the number of studies showing that genetic factors play an important role in different neurological and psychiatric diseases is increasing [76]. Brain imaging measures used in genetic studies should ideally be highly heritable and be genetically related to a biological process affected by genetic variants, such as a disease process [14], [76]–[78]. A greater understanding of such genetic factors might lead to the development of more effective, individually tailored rehabilitation strategies that considers differences in patterns of compensatory plasticity.

## Supporting Information

Figure S1 A) Non-thresholded contrast between encoding new faces vs. viewing a repeated face. Regions that were active in the encoding phase depicted in yellow whereas areas deactivated during the encoding phase are shown in blue. B) Brain areas representing the contrast between encoding new faces in front of viewing a repeated face (thresholded at z = 2.3). The maps depicted in red were the ones used for cortical thickness analysis. rh = right hemisphere; lh = left hemisphere.
(TIF)

Table S1 **ε4 carriers and noncarriers comparison (whole brain analysis).**
(DOCX)

Table S2 **ROI of functional activation.**
(DOCX)

Table S3 **Brain volumetric measurements.**
(DOCX)
