# Correction: APOE Status Modulates the Changes in Network Connectivity Induced by Brain Stimulation in Non-Demented Elders

**DOI:** 10.1371/annotation/fe36cdbc-5ad0-40d4-8289-fe78d2011ca4

**Published:** 2013-10-17

**Authors:** Cleofé Peña-Gomez, Cristina Solé-Padullés, Imma C. Clemente, Carme Junqué, Núria Bargalló, Beatriz Bosch, José Luis Molinuevo, Josep Valls-Solé, Alvaro Pascual-Leone, David Bartrés-Faz

Multiple figure titles and legends were accidentally switched. The correct order for the figure legends can be viewed here: http://www.plosone.org/corrections/pone.0051833.001.cn.docx

