# Supplementary figures and images for: APOE Status Modulates the Changes in Network Connectivity Induced by Brain Stimulation in Non-Demented Elders
Source: PLoS One. 2012 Dec 19;7(12):e51833. doi: 10.1371/journal.pone.0051833 (PMC3526481; doi:10.1371/journal.pone.0051833)

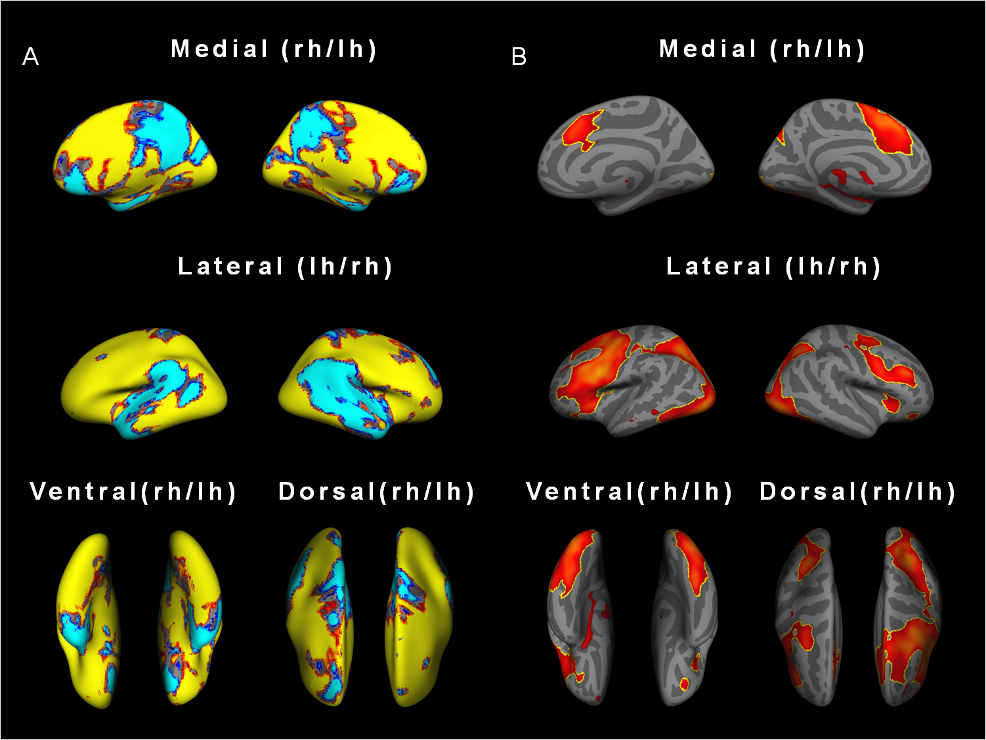

Supplement: Figure S1 — A) Non-thresholded contrast between encoding new faces vs. viewing a repeated face. Regions that were active in the encoding phase depicted in yellow whereas areas deactivated during the encoding phase are shown in blue. B) Brain areas representing the contrast between encoding new faces in front of viewing a repeated face (thresholded at z = 2.3). The maps depicted in red were the ones used for cortical thickness analysis. rh = right hemisphere; lh = left hemisphere. (TIF) [file pone.0051833.s001.tif]
