# Supplementary material for: APOE Status Modulates the Changes in Network Connectivity Induced by Brain Stimulation in Non-Demented Elders
Source: PLoS One. 2012 Dec 19;7(12):e51833. doi: 10.1371/journal.pone.0051833 (PMC3526481; doi:10.1371/journal.pone.0051833)
Supplement: Table S1 — ε4 carriers and noncarriers comparison (whole brain analysis). (DOCX) [file pone.0051833.s002.docx]

**Table S1. ε4 carriers and noncarriers comparison (whole brain analysis).**

| Hemisphere | Size(mm^2)^ | Talairach  Coordinates  x y z | CWP | NVtxs | Area |
| --- | --- | --- | --- | --- | --- |
| RH | 2109.64 | 24.4 -35.3 50.6 | 0.02 | 4981 | Superiorparietal* |
| RH | 1986.74 | 23.9 -60.6 21.4 | 0.03 | 3294 | Precuneus* |

LH=left hemisphere. RH=right hemisphere. mm=millimeters. CWP=Cluster-wise probability. NVtx=Number of vertex. *Areas where ε4-noncarriers had increased thickness in comparison with ε4-carriers.
