# Supplementary material for: APOE Status Modulates the Changes in Network Connectivity Induced by Brain Stimulation in Non-Demented Elders
Source: PLoS One. 2012 Dec 19;7(12):e51833. doi: 10.1371/journal.pone.0051833 (PMC3526481; doi:10.1371/journal.pone.0051833)
Supplement: Table S2 — ROI of functional activation. (DOCX) [file pone.0051833.s003.docx]

**Table S2. ROI of functional activation.**

| **Brain volumetry** | **APOE ɛ4-noncarriers** | **APOE ɛ4-carriers** | **F/p** |
| --- | --- | --- | --- |
| **CTh_ROI_LH** | 2.45 (±0.09) | 2.44(±0.12) | 0.04/0.83 |
| **CTh_ROI_RH** | 2.41(±0.08) | 2.43(±0.13) | 0.02/0.88 |

CTh_ROI_LH= Roi of cortical thickness regarding the left hemisphere. CTh_ROI_RH= Roi of cortical thickness regarding the right hemisphere.
