# Supplementary material for: APOE Status Modulates the Changes in Network Connectivity Induced by Brain Stimulation in Non-Demented Elders
Source: PLoS One. 2012 Dec 19;7(12):e51833. doi: 10.1371/journal.pone.0051833 (PMC3526481; doi:10.1371/journal.pone.0051833)
Supplement: Table S3 — Brain volumetric measurements. (DOCX) [file pone.0051833.s004.docx]

**Table S3. Brain volumetric measurements.**

| **Brain volumetry** | **APOE ε4-noncarriers** | **APO ε4-carriers** | **F/p** |
| --- | --- | --- | --- |
| **BrainSegVol** | 1116.59 (±74.35) | 1086.81(±105.70) | 0.09/0.76 |
| **ICV** | 1426.28 (±83.34) | 1439.21(±95.03) | 0.75/0.39 |
| **Left grey matter** | 240.06(±21.78) | 231.66(±24.51) | 0.09/0.76 |
| **Right grey matter** | 238.18(±22.29) | 228.93(±25.26) | 0.12/0.72 |
| **Left white matter** | 207.35(±18.28) | 199.57(±28.92) | 0.84/0.37 |
| **Right white matter** | 206.38(±18.23) | 199.57(±28.98) | 0.14/0.70 |
| **Left hippocampus** | 4.14(±0.62) | 4.02(±0.66) | 0.97/0.75 |
| **Right hippocampus** | 3.98(±0.58) | 3.96(±0.63) | 0.15/0.70 |
| **CSF** | 1346.91(±0.23) | 1470.11(±0.19) | 1.68/0.22 |

Values denote mean (± SD) units are in cm^3^. ICV=Intracraneal Volume. CSF= Cerebro spinal fluid.
